# Nanotherapy and Reactive Oxygen Species (ROS) in Cancer: A Novel Perspective

**DOI:** 10.3390/antiox7020031

**Published:** 2018-02-22

**Authors:** Peter Brenneisen, Andreas S. Reichert

**Affiliations:** Institute of Biochemistry and Molecular Biology I, Medical Faculty, Heinrich-Heine-University Düsseldorf, 40225 Düsseldorf, Germany; reichert@hhu.de

**Keywords:** tumor-stroma interaction, reactive oxygen species (ROS), chemotherapeutics, nanoparticle, cerium oxide, mitochondria, combinational therapy

## Abstract

The incidence of numerous types of cancer has been increasing over recent years, representing the second-most frequent cause of death after cardiovascular diseases. Even though, the number of effective anticancer drugs is increasing as well, a large number of patients suffer from severe side effects (e.g., cardiomyopathies) caused by these drugs. This adversely affects the patients’ well-being and quality of life. On the molecular level, tumor cells that survive treatment modalities can become chemotherapy-resistant. In addition, adverse impacts on normal (healthy, stromal) cells occur concomitantly. Strategies that minimize these negative impacts on normal cells and which at the same time target tumor cells efficiently are needed. Recent studies suggest that redox-based combinational nanotherapies may represent one option in this direction. Here, we discuss recent advances in the application of nanoparticles, alone or in combination with other drugs, as a promising anticancer tool. Such novel strategies could well minimize harmful side effects and improve patients’ health prognoses.

## 1. Redox-Based Combinational Nanotherapy: A Novel Anticancer Strategy

Numerous approaches in anticancer therapy focus largely on tumor cells, and solely address the question of how anticancer drugs affect tumor cell survival and metastasis [1,2,3,4]. The bilateral communication between cancer cells and the tumor microenvironment—the so-called tumor-stroma interaction—is usually neglected [5,6,7]. There is increasing evidence that stromal components actively take part, for example, in tumor invasion, neoangiogenesis, metastasis, and development of drug resistance [8,9]. Aside from components of the extracellular matrix and soluble factors, cellular components such as immune cells, endothelial cells, and cancer-associated fibroblast are involved in the interplay with tumor cells [10]. In the battle against cancer, traditional options such as surgery, radiation therapy and/or chemotherapy are used, but often result in harmful side effects on normal (healthy, stromal) cells, thus affecting the whole organism and carrying the risk of late secondary cancer.

Many of the well-used “classical” chemotherapeutic agents show a very effective cytostatic/cytotoxic function based on their broad range of chemical efficacy. Examples include cisplatin or anthracyclin derivates such as doxorubicin (adriamycin) or daunorubicin [11], among others. In fact, those drugs act rather unspecifically, and thus they adversely influence healthy/stromal cells and tissues. Alopecia, vomiting, nausea, mucositis, cardiomyopathies, and nephrotoxicity are some of the severe side effects to the patients to name but a few. Furthermore, the occurrence of drug resistance and prevention of overcoming the blood-brain barrier (BBB) can be problematic with these drugs as well [12,13,14,15,16]. Owing to this detrimental impact of those chemotherapeutics on mental and physical well-being and quality of life for cancer patients, it is mandatory in the near future to develop novel and/or supplemental or combinational therapies which, on the one hand, lower harmful effects; and on the other hand, improve lifespan and quality of life of the patients. In that context, a personalized medicine may be of advantage, meaning specific drugs, inhibitors and antibodies among others used for treatment tailored to a single patient’s genetic profile. But on the other hand, the improved health outcome for such patients may only come at a high cost for the patient and/or for the health insurance companies, as it is presently still very costly to screen patients for such specialized treatments and to produce medicine that targets only individuals or narrow groups of patients. Even though most analytical studies describe many personalized medicine tests as being very cost-intensive, a few studies have found that a personalized medicine may save costs [17,18,19,20]. In conclusion, more evidence on the value of such a novel medicine is needed in the context of assessing genomic priorities, cost recovery and ethical aspects such as its availability for all groups of a population. But before such a personalized medicine can become a daily routine for all, possibly resulting in a “transparent man”, another option in anticancer therapy may be able to modify already existing therapeutic approaches and combine them in novel combinational therapies, allowing (i) a direct transit of drugs to the tumor location; (ii) the killing of the tumor cells; and (iii) the protection of healthy/stromal cells against the dominating influence of the tumor cells on the stromal cells and against the damaging effect of the anticancer drug. Ultimately, this would result in fewer side effects and a better prognosis. Before discussing such a promising combinational approach, it is important to ask the question: “What is the difference between a tumor cell and its normal counterpart or stromal cell?” Aside from altered signaling pathways and metabolic processes, which differ from tumor type to tumor type, an increased level of reactive oxygen species (ROS) compared to normal cells is common to almost all tumor cells [21,22,23]. This is proposed as a possible common target for therapeutic approaches [24,25,26].

**Figure 1:**
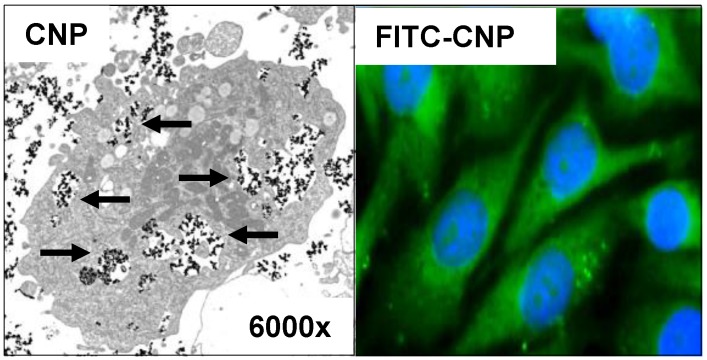
**Cellular uptake of cerium oxide nanoparticles (CNP)** Squamous tumor cells of the skin were treated with 150 µM CNP or fluorescein isothiocyanate (FITC)-labeled CNP for 24 h. Data of studies with trans electron microscopy (**left**) indicate uptake of CNP (see arrows). Studies with FITC-CNP (green fluorescence) show distribution of CNP in the cytosol, while there appears no green fluorescence (or merged cyan fluorescence) of the nuclei (blue, 4′,6-Diamidin-2-phenylindol (DAPI)-staining).

Depending on their reactivity and localization, ROS are involved in physiological (termed oxidative eustress) and pathophysiological processes (termed oxidative stress) [27,28]. One major source of increased cellular ROS levels is dysfunctional mitochondria [29]. However, increased ROS levels and, in consequence, an altered redox status of the cell provide a specific vulnerability of cancer cells, which can be used for therapeutic approaches [30]. Recently, two opposing redox status modulating therapies have been tested in clinical trials. In the first approach, antioxidants are used to lower the content of ROS in tumor cells, subsequently inducing cell cycle arrest and, lastly, apoptosis [31,32]. The other prooxidative concept aims to increase the ROS level of cancer cells to an extent that exceeds their survival strategies, and in the end results in an increased apoptotic rate as well [24,26,33]. Normally, the imbalance of the prooxidative and antioxidative systems towards higher ROS levels in cancer cells is still maintained below a cytotoxic threshold. Impairment of the cellular antioxidant system or treatment with exogenous ROS generating agents [11] might exceed a certain ROS threshold, thus resulting in detrimental oxidative stress. That stress can apparently not be compensated by the defense systems of cancer cells, finally leading to cytotoxic effects and lastly to apoptosis or necrosis. In contrast, healthy cells are often able to counteract increased ROS levels by their endogenous antioxidant systems [34]. In addition to their cytostatic effects on tumor cells, chemotherapeutical drugs, such as the above-mentioned anthracyclins, may act as such prooxidants. Nevertheless, both tumor and normal (healthy, stromal) cells are damaged by such compounds, resulting in severe side effects. This is often accompanied by enhanced drug-mediated ABC- (ATP-binding cassette) expression in some tumor types [35,36]. Also a doxorubicin induced increase of autophagy associated with an increase in survival and a lowered apoptosis rate was observed [37,38], which may result in increased tumor resistance. Therefore, the clinical use and outcome of these “dirty drugs” seems to be limited in terms of application [39]. But what can be done? Recently, molecules acting on ROS production, disrupting mitochondrial function and, finally, resulting in apoptosis of cancer cells are discussed. In that context, manganese (Mn)-superoxide dismutase (SOD) mimics [40], copper(II) phenanthroline metallopeptides [41], selenium-containing quinone-based triazoles [42] or organoiridium complexes [43] are some examples of molecules/catalysts being promising anticancer agents. In nanotherapy, the medical application of nanotechnology, promising approaches are currently developed and partly already tested in clinical trials [44]. One of the promising applications in nanomedicine is the use of nanoparticles (<100 nm in size) as carrier system for drug delivery to specific cellular targets or organs, for example the transport to mitochondria [45] or to the brain [46]. Here are just four of the many examples that could be mentioned, bringing to mind the tremendous potential of nanocarriers in anticancer therapies. In an interesting approach using a rat glioma model, the acidic tumor microenvironment was exploited to transport pH-sensitive doxorubicin-loaded PEGylated-gold nanoparticles to the tumor site, resulting in rapid release of doxorubicin at low pH and in an increase of ROS level [47]. Recently, doxorubicin-loaded lipid nanoparticles were tested for topical treatment of skin cancer. Cytotoxicity data with murine melanoma cells and the histological analysis of melanoma induced Balb/C mice showed promising results with those nanoparticles [48]. Furthermore, encapsulation of the platinum(IV) prodrug mitaplatin in block copolymer nanoparticles resulted in an increase in drug circulation time in blood and in a controlled drug release [49]. Due to their nontoxic nature, carbon-based nanoparticles (carbon dots, carbon nanotubes) are used as platform to deliver drugs such as doxorubicin and gemcitabine to the tumor site [50]. However, most of these nanoparticles still face tremendous challenges regarding their limited biocompatibility and subsequent low cytotoxicity in some types of tumor cells [45,51]. Meanwhile, the application of nanoparticles, which themselves act directly as pharmacological substances (nanopharmaceutical), appears to be another therapeutic possibility for the treatment of some cancer types. In this context, especially the lanthanide cerium in the form of polymer-coated or polyacrylate-stabilized cerium oxide (CeO_2_/Ce_2_O_3_) nanoparticles (CNP, nanoceria), shows promising properties regarding a redox-modulatory and enzyme-like activity. Due to oxygen vacancies on the surface of CNP and the ability to autocatalytically switch between the oxidation states IV (Ce^4+^) and III (Ce^3+^), CNP function as a pro- or antioxidant based on the microenvironment [52,53,54,55]. Such nanopharmaceuticals can be used in a redox-based chemotherapy as “stand-alone drugs”, or in combination with classical chemotherapeutical agents, like the successful approaches with doxorubicin (see below). In recent years, an increasing number of articles have dealt with nanoceria as an antioxidant-detoxifying superoxide, hydrogen peroxide, and peroxynitrite and, therefore, as having a beneficial effect on different cells and tissues in vitro [56,57,58,59,60]. For example, CNP protect human dermal fibroblasts from the redox cycler paraquat (PQ) initiated increase of superoxide (O_2_^−^) level [56]. The uptake of nanoceria results in a subcellular distribution (Figure 1). Interestingly, it seems that CNP do not enter the nucleus and, thus, exert no genotoxicity which is beneficial for normal, healthy cells [11]. On the other hand, CNP were shown by others to be present in the nucleus and, notably, also to be co-localized with ROS-producing mitochondria [61].

**Scheme 1:**
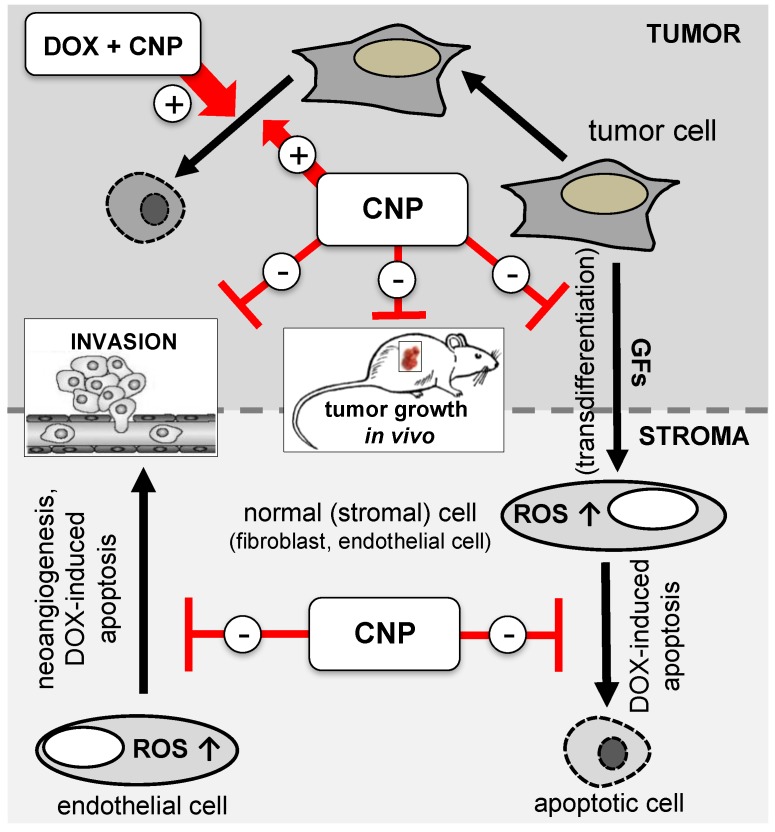
**Bifunctional role of cerium oxide nanoparticles (CNP) in tumor-stroma interaction** CNP prevent (−) tumor cell-derived growth factor (GF)-dependent modulation of normal (stromal) cells (e.g., transdifferentation, neoangiogenesis) and mediate their protection from doxorubicin (DOX)-initiated apoptotic cell death. Furthermore, CNP lower tumor invasion and tumor growth in vivo. In contrast, CNP induce (+) reactive oxygen species (ROS)-mediated cell death in the studied tumor cells and enhance the apoptotic rate in a combinational approach with DOX [11,58,60].

Hirst and coworker detected CNP around the mitochondria which subsequently scavenge mitochondria-generated ROS in normal murine macrophages [62]. Furthermore, nanoceria were shown to protect mitochondria from mitochondrial fragmentation (fission) in primary skin fibroblasts [63]. Nanoceria caused an increased release of cytochrome *c* from mitochondria into the cytosol in tumor cells [64]. Overall, CNP appear to act inter alia via modulating mitochondrial function and/or mitochondrial ROS generation. In skin cancer and glioma cells, exogenously added CNP with a defined size have been shown to act as a cell-killing and anti-invasive agent via increasing the level of distinct reactive oxygen species like hydrogen peroxide. In contrast, the same concentration of CNP is nontoxic in stromal (healthy) fibroblasts or endothelial cells [64,65,66], suggesting a bifunctional role of nanoceria in tumor-stroma (Scheme 1). In this context, in a combinational approach with the chemotherapeutical drug doxorubicin, CNP enhance the ROS-mediated toxicity of doxorubicin in different cancer cells, while CNP exhibit some protection against doxorubicin mediated cell death in human dermal fibroblasts and rat cardiomyocytes [11,67]. In addition to the increasing number of publications with in vitro data, more and more in vivo data are available today. To quote only three, the infiltration of immune cells and the expression of proinflammatory cytokines, which goes hand-in-hand with ventricular dysfunction and dilatation, can be significantly lowered by the use of cerium oxide nanoparticles, studied on a murine model of cardiomyopathy [68]. Our studies on a xenograft mouse model show that CNP significantly lower tumor growth and invasion and, furthermore, inhibit some processes of neoangiogenesis [64]. Recently, folic acid-tagged cerium oxide nanoparticles have been shown to increase the cellular nanoparticle internalization and inhibit cell proliferation of an ovarian cancer cell line and significantly lower the tumor burden of such mouse xenografts [69]. Aside from the use in cancer research, it was shown in animal models that mitochondria-targeted cerium oxide nanoparticles protect against amyloid beta (Aβ) induced mitochondrial fragmentation and cell death in a model for Alzheimer’s disease [70,71].

Aside from the fact that a planned personalized (anticancer) medicine in the future still presents many questions and challenges, it appears that other possible anticancer strategies are already available, dealing with major challenges such as killing the tumor cells without damaging normal (healthy, stromal) cells, and lowering harmful effects to improve prognosis and quality of life of the patient. In particular, a ROS-modulating nanotherapy could play an important role in the 21st century. A nanoparticle-based anticancer therapy with either specific nanocarriers or nanopharmaceuticals, which have tremendous potential in a combination therapy, could support classical anticancer strategies such as the use of ROS-producing anthracyclins. Such a nanoparticle-based combination therapy could fulfill the above-mentioned anticancer guideline of killing tumor cells and leaving normal cells intact with less side effects. Certainly, other combinational anticancer approaches, which are independent of the use of nanoparticles, but also depend on modulation of the ROS level, will be discussed to be promising for an anticancer therapy [72].

## 2. Conclusions

In conclusion, special nanoparticles such as cerium oxide nanoparticles (nanoceria) appear to be a promising and powerful tool for the development of a nanoparticle-based redox-directed combinational anticancer therapy in the (near) future that is accessible to all classes of society. Such an approach may combine the high efficacy of chemotherapeutical drugs, such as the anthracyclines, with the drug supporting effect (on tumor cells) and protecting potential (on normal cells) of bifunctionally active nanoparticles. However, more data from animal models and current and future clinical trials will show whether the nanomedicine will meet the expectations generated by the recent promising results in cancer research as well as in other fields including neurodegeneration.
